# Study on Carboxymethylation Modification of Konjac Gum and Its Effect in Drilling Fluid and Fracturing Fluid

**DOI:** 10.3390/gels11100792

**Published:** 2025-10-02

**Authors:** Yongfei Li, Pengli Guo, Kun Qu, Weichao Du, Yanling Wang, Gang Chen

**Affiliations:** 1 Shanxi Province Key Laboratory of Environmental Pollution Control and Reservoir Protection Technology of Oilfields, Xi’an Shiyou University, Xi’an 710065, China; 17645179148@163.com (P.G.); w13966930670@163.com (K.Q.); duweichao@xsyu.edu.cn (W.D.); 2 Engineering Research Center of Oil and Gas Field Chemistry, Universities of Shaanxi Provence, Xi’an Shiyou University, Xi’an 710065, China; 3 College of Petroleum Engineering, China University of Petroleum (East China), Qingdao 266580, China; wangyl@upc.edu.cn

**Keywords:** Konjac gum, modification, hydrogel, viscosity

## Abstract

With the continuous progress and innovation of petroleum engineering technology, the development of new oilfield additives with superior environmental benefits has attracted widespread attention. Konjac glucomannan (KGM) is a natural resource characterized by abundant availability, low cost, biodegradability, and environmental compatibility. Konjac gum easily forms a weak gel network in water, but its water solubility and thermal stability are poor, and it is easily degraded at high temperatures. Therefore, its application in drilling fluid and fracturing fluid is limited. In this paper, a method of carboxymethyl modification of KGM was developed, and a carboxymethyl group was introduced to adjust KGM’s hydrogel forming ability and stability. Carboxymethylated Konjac glucomannan (CMKG) is a water-soluble anionic polysaccharide derived from natural Konjac glucomannan. By introducing carboxymethyl groups, CMKG overcomes the limitations of the native polymer, such as poor solubility and instability, while retaining its safe and biocompatible nature, making it an effective natural polymer additive for oilfield applications. The results show that when used as a drilling fluid additive, CMKG can form a stable three-dimensional gel network through molecular chain cross-linking, significantly improving the rheological properties of the mud. Its unique gel structure can enhance the encapsulation of clay particles and inhibit clay hydration expansion. When used as a fracturing fluid thickener, the viscosity of the gel system formed by CMKG at 0.6% (*w*/*v*) is superior to that of the weak gel system of KGM. The heat resistance/shear resistance tests confirm that the gel structure remains intact under high-temperature and high-shear conditions, meeting the sand-carrying capacity requirements for fracturing operations. The gel-breaking experiment shows that the system can achieve controlled degradation within 300 min, in line with on-site gel-breaking specifications. This modification process not only improves the rheological properties and water solubility of the CMKG gel but also optimizes the gel stability and controlled degradation through molecular structure adjustment.

## 1. Introduction

The global expansion of oil and gas exploration into deep formations, high-temperature/high-pressure environments, and ecologically sensitive zones has driven a paradigm shift in drilling and fracturing fluid additives—transitioning from conventional synthetic materials to green intelligent alternatives [1]. Traditional synthetic oilfield additives often contain heavy metals (e.g., Pb, As, Hg, Zn), toxic organic agents, and complex components, posing significant risks to reservoir integrity, groundwater safety, and ecological balance, while compromising hydrocarbon quality [2,3]. This underscores the urgent need to develop multifunctional additives integrating high-efficiency, eco-compatibility, and cost-effectiveness as a critical pathway for sustainable energy extraction. In the late 1950s, U.S. researchers pioneered the use of guar gum—a galactomannan-rich plant gum, as the primary component of drilling fluids, marking the first application of plant-derived polymers in this field [4]. Early drilling fluids evolved from rudimentary “clay–water mixtures” to advanced formulations, including dispersed (fine and coarse), inhibitive, deep-well, and low-solid drilling fluids.

By the 1970s, plant gums and their derivatives were proposed as alternatives to diesel-based viscous fluids for fracture initiation and propagation in fracturing operations [5]. A milestone emerged in the 1990s with the successful deployment of plant-gum additives in the highly water-sensitive shale formations of the Gulf of Mexico, demonstrating their dual functionality in fluid loss control and lubrication [6].

Konjac gum is a naturally sourced plant-derived material and is environmentally benign. It is a water-soluble non-ionic polysaccharide primarily composed of β-D-mannose and β-D-glucose in specific ratios [7]. In its unique molecular structure, functional groups such as primary and secondary hydroxyl groups and acetyl groups are like “active sites” for chemical reactions, providing an ideal site for graft copolymerization reactions [8]. When Konjac gum dissolves in water, its molecules are tightly intertwined with water molecules through intermolecular forces such as dipole interactions and hydrogen bonds, forming a gel-like solution with high viscosity [9,10]. This solution exhibits typical pseudoplastic fluid characteristics—its viscosity decreases when stirred externally and returns to its original state when left standing. This property endows it with excellent suspension and rock powder carrying capabilities. What is more noteworthy is that Konjac gum originates from natural plant resources, conforming to the concept of green environmental protection. When combined with metal ions such as K^+^ and Ca^2+^, or heated to above 60 °C, the hydrogen bond interaction between Konjac gum molecules is enhanced, and double helix aggregation occurs. Depending on specific conditions, thermo-irreversible or reversible hydrogels can be formed. Therefore, Konjac gum has received extensive attention and application. In particular, Konjac gum is used in drilling fluids and fracturing fluids. Zhang Jie et al. [11] studied the polysaccharide SJ drilling fluid and used it as a water-based drilling fluid additive. At concentrations ranging from 1% to 6%, it demonstrated good inhibition of mud shale hydration and expansion, with a temperature resistance limit of up to 140 °C. It was successfully applied as an environmentally friendly oilfield chemical in the Jiangsu Oilfield. Xue et al. [12] developed two novel polymers that maintain good rheological properties and fluid loss control in drilling fluid systems at 230 °C. However, the presence of high water-insoluble content, slow dissolution rate, and poor fluidity in Konjac powder prevents its direct utilization. Consequently, chemical modifications of plant gums are required to enhance their applicability as oilfield additives.

Konjac gum modification primarily utilizes two approaches: crosslinking modification and grafting modification [13]. Crosslinking modification encompasses both physical and chemical methods: Physical crosslinking promotes the entanglement of molecular chains through physical treatment, forming a spatial network structure, thereby enhancing the stability and hydrolysis resistance of molecules. Chemical crosslinking, on the other hand, uses modifiers such as aldehydes, boron compounds, and epichlorohydrin to react with hydroxyl groups on the molecules through etherification or esterification to form chemical bonds, resulting in derivatives that optimize the molecular chain structure, thereby improving key properties such as temperature resistance, viscosity enhancement, and fluid loss control in oilfield chemical operations [14]. Grafting modification, conversely, involves modifiers forming etherification or esterification bonds with the hydroxyl groups to introduce new functional groups, thereby altering the molecular architecture; this is further classified into cationic modification (introducing positively charged groups), anionic modification (introducing negatively charged groups) and non-ionic modification (introducing electrically neutral groups) based on the electrical properties of the incorporated functional groups. Despite the widespread application of these traditional modification methods to enhance the performance of natural plant gums, such as KGM, in drilling and fracturing systems, current modification strategies often focus on improving a single property, failing to address the interwoven challenges faced by natural colloids: suboptimal solubility, process-related pollution, and inadequate adaptability to extreme environments [15,16]. In particular, KGM is frequently plagued by solubility limitations and environmental concerns during conventional modification processes. The strong intermolecular hydrogen bonds of KGM lead to its low solubility in water at room temperature, requiring the assistance of high-temperature conditions. This not only increases energy consumption but may also damage the molecular structure, cause uneven reactions, and affect the performance of the products. Meanwhile, the use of aldehyde crosslinking agents and high-temperature, high-pressure processes in the modification process will bring about environmental problems such as wastewater pollution and high energy consumption. In addition, the biodegradability of some modified products deteriorates, aggravating the environmental burden.

Therefore, to address the long-standing efficiency–environment conflict of natural colloids in engineering applications, this study proposes a green strategy based on carboxymethylation modification. Centered on carboxymethylation, a modified colloid CMKG was successfully constructed through a reaction in an ethanol/water mixed medium under alkaline conditions. Through orthogonal experiments, the optimal reaction conditions for the modification process were determined (temperature: 60 °C, duration: 4 h, NaOH: 1.5 g, DCA: 3.0 g). This eco-friendly modification strategy significantly improves both the solubility and multifunctionality of the Konjac-derived product. CMKG improved rheological control (418.2% viscosity enhancement at 0.3% *w*/*v*), reduced water-insoluble content (≤1.2%), and elevated environmental compatibility while reducing dosage requirements. Compared to conventional additives, CMKG achieved an 18% cost reduction and a 30% improvement in biodegradability. It has driven the transition toward green, efficient oilfield chemicals, enabling sustainable hydrocarbon extraction from deep and environmentally sensitive reservoirs.

## 2. Results and Discussion

### 2.1. Modification of KGMs

**Figure 1:**
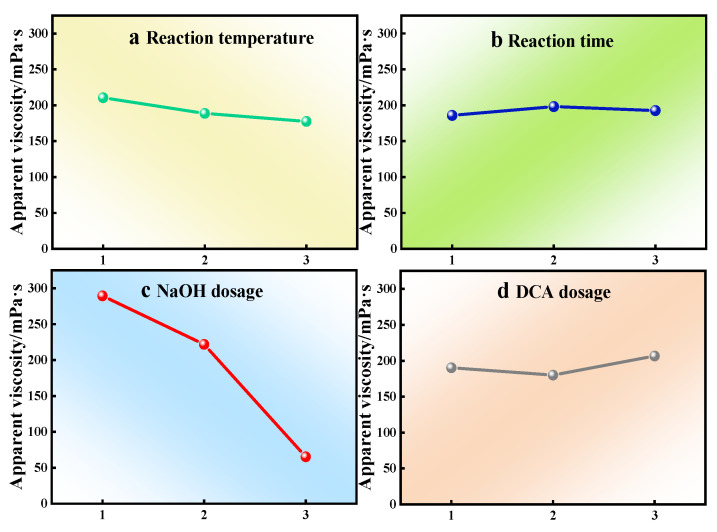
Main effect diagram of the mean value (apparent viscosity) of the orthogonal test.

**Figure 2:**
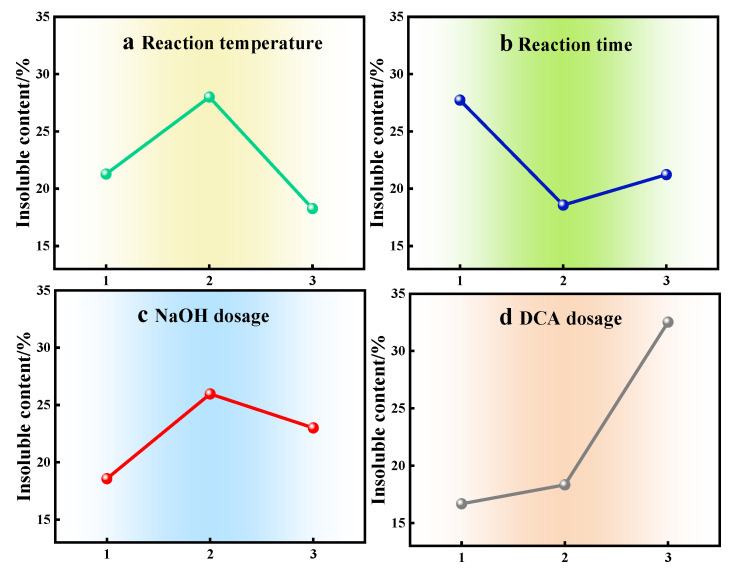
Main effect plot of orthogonal test means (insoluble matter content).

A four-factor, three-level orthogonal experiment was implemented to investigate the effects of critical processing parameters—reaction temperature (a), reaction duration (b), NaOH dosage (c), and modifying agent DCA dosage (d)—on the performance of modified products. The apparent viscosity of the gum and the water-insoluble content were primarily compared before and after carboxymethylation modification to evaluate the performance. Orthogonal experimental results are summarized in Table 1, while range analysis outcomes detailing the hierarchical influence of factors and optimal parameter combinations are presented in Table 2 and Table 3. Mean effect plots (Figure 1 and Figure 2) further illustrate the relationship between factor levels and response variables. Range analysis methodology was systematically applied to discern the dominant factors governing modification efficiency and establish priority rankings among variables, thereby enabling the determination of optimal modification conditions through multi-criteria optimization.

Reaction temperature (a), reaction time (b), NaOH dosage (c), and DCA dosage (d) all demonstrate statistically significant impacts on the viscosity of modified products with quantifiable trends. The magnitude order of influence is c > a > d > b (Table 3 and Figure 1). This indicates that NaOH dosage (c) exerts the most pronounced influence on the CKGM, followed by reaction temperature (a) and DCA dosage (d), while reaction duration (b) shows the least impact. Notably, incremental increases in sodium hydroxide dosage and reaction temperature correlate strongly with enhanced apparent viscosity of the modified product, suggesting a dosage- and temperature-dependent relationship. To achieve maximal viscosity, the optimized modification parameters were identified as sodium hydroxide dosage 0.1% (*w*/*w*), reaction temperature 30 °C, DCA dosage 1.0% (*w*/*w*), and reaction duration 4 h. These conditions were derived through rigorous range analysis, which prioritized factor contributions and validated the interplay between process variables and target performance metrics.

The order of influence of the four factors on the water insolubility of the product is determined as follows: DCA dosage (d) > reaction temperature (a) > reaction time (b) > NaOH dosage (c) (Table 1 and Figure 2). This indicates that the DCA additive amount has the most significant impact on the water insolubility of CMKG, followed by reaction temperature, reaction time, and sodium hydroxide additive amount, which shows the least influence. Taking into comprehensive consideration the effects of these four factors on both the apparent viscosity and water insolubility of the product, the optimal reaction conditions were determined as follows: reaction temperature 30 °C, reaction duration 2 h, sodium hydroxide additive amount 0.1 wt%, and DCA additive amount 1.0 wt%.

### 2.2. Characterization Results

#### 2.2.1. FTIR Analysis

**Figure 3:**
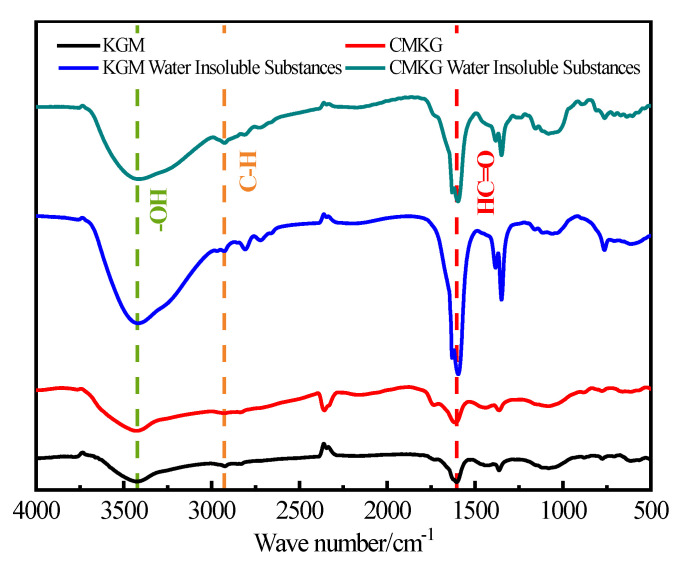
Infrared analysis spectrum.

Under the preferred conditions, the carboxymethyl modification reaction of KGM was carried out to prepare CMKG. Infrared spectroscopy analysis was conducted (Figure 3). The main absorption peaks of KGM and CMKG are highly similar. The characteristic hydrogen-bonded peaks of polyhydroxyl (-OH) groups appear in the range of 3360–3450 cm^−1^, while the C-H stretching vibration absorption peaks of aliphatic chains are observed at 2850–2925 cm^−1^. The carbonyl group (HC=O) stretching vibration absorption at 1728–730 cm^−1^ corresponds to the acetyl groups on the KGM molecular chain. The absorption peaks at 876 cm^−1^ (β-D-glycosidic bond configuration) and 811 cm^−1^ (pyranose ring vibration) are retained in the infrared spectra of both KGM and CMKG, indicating that the primary structure of the KGM backbone remains unchanged before and after carboxymethyl modification. On the other hand, two new absorption peaks emerge in the CMKG spectrum—1569 cm^−1^ and 1416 cm^−1^—which are characteristic of carboxylate groups (-COO^−^), confirming the successful introduction of carboxymethyl groups onto the KGM molecular chain, thereby achieving the desired modification effect. Furthermore, the infrared spectra of the water-insoluble components before and after modification show no significant differences, suggesting that the carboxymethylation modification has minimal impact on the water-insoluble substances in KGM.

#### 2.2.2. DSC Analysis

**Figure 4:**
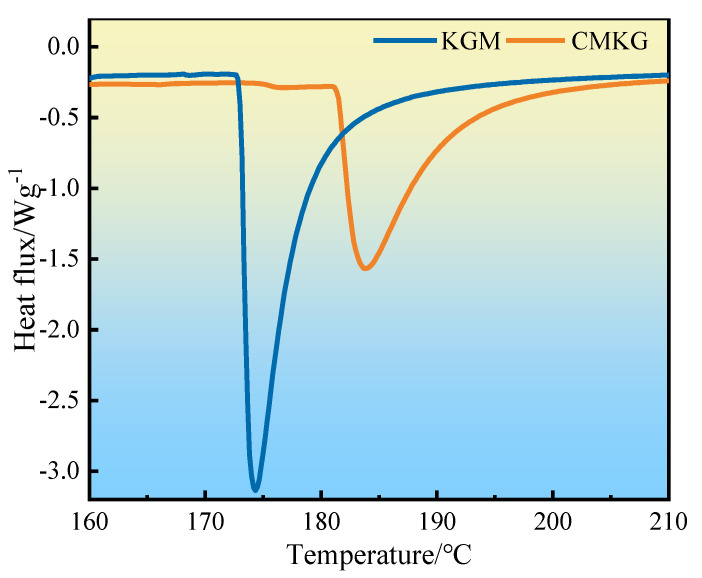
DSC analysis diagram of KGM and CMKG. Note: The DSC curve excludes the data of the initial unstable stage.

The DSC thermograms of KGM and CMKG are depicted in Figure 4. KGM and CMKG exhibit gradual endothermic behavior as the temperature increases. The KGM displays an endothermic peak starting at 172 °C, with the phase transition absorption peak centered around 175 °C, while CMKG shows an endothermic peak at 180 °C and a phase transition peak at 185 °C. Compared to KGM, both the endothermic and phase transition peaks of CMKG shift to higher temperatures, indicating that partial thermal decomposition of some compounds in the modified product begins at 175 °C. This phenomenon may directly impact the thermal resistance of the CMKG when applied to water-based drilling fluids.

#### 2.2.3. TG Analysis

**Figure 5:**
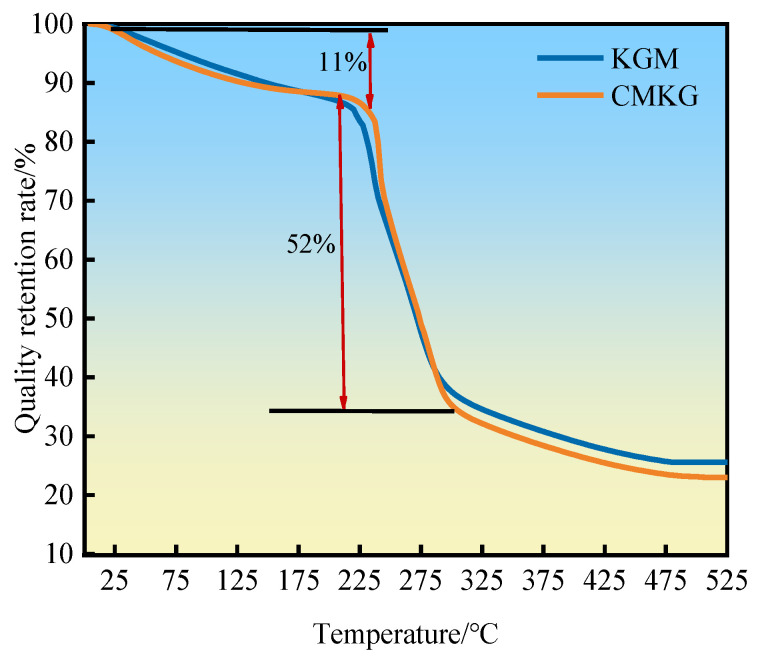
TGA of KGM and CMKG.

Due to the presence of substantial free water and bound water in KGM and CMKG, as the temperature rises, evaporation gradually occurs, resulting in a change in mass. Therefore, the mass–temperature relationship is further studied. The thermogravimetric (TGA) curves of KGM and CMKG are shown in Figure 5. As observed, the gum powders undergo stepwise decomposition with increasing temperature. Below 105 °C, the TGA curves of KGM and CMKG are nearly identical, with KGM exhibiting a slightly higher mass retention rate compared to the carboxymethylated product, attributed to the evaporation of free water. In the range of 180–220 °C, the mass retention rate gradually decreases as temperature rises, indicating the onset of thermal decomposition of compounds in the gum powders. Between 220 and 300 °C, the powders enter a distinct weight-loss phase, with a sharp decline in mass retention, suggesting extensive decomposition of certain compounds in both KGM and CMKG. Combined with the DSC curves, this confirms that the decomposition temperature of CMKG compounds occurs around 200 °C.

#### 2.2.4. Elemental Analysis

The elemental composition (C, H, N, S, O) of KGM and CMKG was determined using a VarioMICRO elemental analyzer, and the results are summarized in Table 4. Significant variations in the content of C, H, N, S, and O were observed before and after carboxymethylation modification. For the CMKG, the elemental compositions were measured as 39.37% C, 6.41% H, 0.18% N, 0.31% S, and 53.72% O. Compared to the KGM, the carbon (C) content increased by 1.57%, while the oxygen (O) content decreased by 1.37%. These changes in C and O content confirm the successful introduction of new functional groups (e.g., carboxymethyl groups) into the KGM molecular chain during modification.

### 2.3. Application of KGM and CMKG in Drilling Fluid

Load oven-dried bentonite into the core mold assembly. Compress the assembly at 10 MPa for 5 min using a hydraulic press to form core discs. Prepare KGM and CMKG solutions at predetermined concentrations. Measure disc swelling in these solutions with a linear swell meter, recording expansion values at timed intervals. Calculate the linear expansion rate of the clay. Performance evaluation of drilling fluid additives, encompassing key indicators such as rheology, filtration loss, lubricity, inhibitory properties, and temperature/salt resistance, constitutes an integral part of developing new additives and their field application. Systematic laboratory testing enables direct assessment of additive efficacy within drilling fluids. CMKG was evaluated with a focus on its viscosity-enhancing capability, filtration loss reduction, temperature resistance, and ability to inhibit clay hydration and swelling, with performance comparisons made against KGM. Through characterization techniques such as FTIR spectroscopy, differential scanning calorimetry (DSC), thermogravimetric analysis (TGA), and elemental analysis, the mechanism of KGM/CMKGM in drilling fluid can be preliminarily discussed.

#### 2.3.1. Drilling Fluid Performance

The fundamental performance parameters of drilling fluid treated with KGM and CMKG were measured at different concentrations (0.05%, 0.10%, and 0.30%) and compared with the base mud. The results are presented in Table 5. The results demonstrate that both KGM and CMKG-treated fluids exhibited enhanced apparent viscosity compared to the base mud, with viscosity increasing proportionally to additive concentration. Notably, CMKG outperformed KGM in viscosity enhancement at equivalent concentrations, achieving viscosity enhancement rates of 418.2% versus 390.0% at 0.3% dosage. This superiority can be attributed to the CMKG, which improves hydrophilicity and enables ionization of polyvalent anions in the drilling fluid. The terminal hydroxyl and ether oxygen groups on CMKG molecules formed hydrogen bonds with clay particle surfaces while coordinating with edge-exposed Al^3+^ ions at broken bond sites, promoting compact adsorption on clay surfaces and thereby amplifying viscosity [17]. Both additives increased plastic viscosity (PV) and yield point (YP). At a 0.05% dosage, KGM and CMKG demonstrated moderate filtration reduction, whereas higher concentrations (>0.05%) paradoxically increased fluid loss. This phenomenon may arise from excessive additive–clay interactions forming stable three-dimensional networks: KGM’s saccharide chains created hydroxyl-water hydrogen-bonded crystalline junction points, while CMKG’s modified structure generated interpenetrating networks [18]. These intertwined architectures induced partial gelation and flocculation in the mud system. Slider friction tests indicate that appropriate KGM and CMKG additions improved the lubricity of filter cakes. Comprehensive evaluation suggests that 0.05% additive concentration optimally enhances drilling fluid performance by balancing rheological enhancement, filtration control, and operational efficiency.

#### 2.3.2. Evaluation of Temperature Resistance Performance

**Figure 6:**
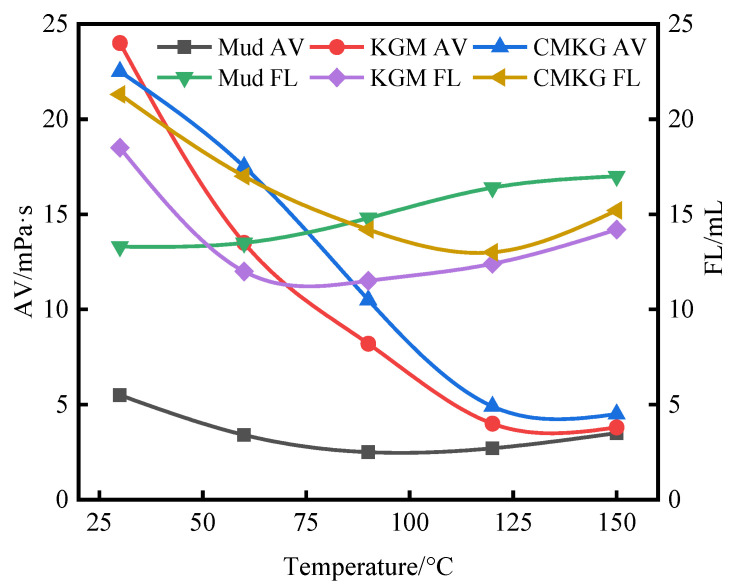
Influence of temperature on the slurry treatment performance of KGM and CMKG.

Drilling fluid samples treated with 0.1% KGM and CMKG, respectively, were prepared, aged for 16 h in a hot rolling oven at temperatures of 30 °C, 60 °C, 90 °C, 120 °C, and 150 °C, and then evaluated for fundamental performance parameters. Comparative results against base mud samples subjected to identical aging conditions are presented in Table 6. Compared with the base mud, the sliding friction coefficient exhibited a slight reduction in the treated systems. The apparent viscosity (AV) and filtration loss (FL) parameters of the 0.1% KGM and CMKG treated muds are plotted in Figure 6. After adding KGM and CMKG, the AV values of treated muds remained consistently higher than those of the base mud across all aging temperatures. However, with increasing temperature, the AV of treated muds progressively decreased, reaching its minimum at 150 °C.

Regarding filtration characteristics, the base mud demonstrated continuously increasing FL with rising temperature, whereas the treated muds exhibited an initial decrease followed by an increase in FL. Notably, the FL of KGM and CMKG treated muds exceeded that of the base mud below 70 °C but became lower thereafter. This phenomenon is attributed to (1) In the initial stage, the incomplete swelling of KGM and CMKG macromolecules interacted with clay particles, creating a more dispersed system that slightly increased FL compared to the base mud [19]. (2) As temperature increased, enhanced molecular motion facilitated the formation of stable cross-linked networks between conventional clay and polymer molecules, thereby reducing FL [20]. (3) With further temperature elevation, intensified molecular motion weakened hydrogen bonding between polymer chains and clay particles, leading to the collapse of the three-dimensional network structure formed by clay-polysaccharide interactions. This structural degradation macroscopically manifested as AV reduction. Concurrently, enhanced interparticle adsorption between clay particles caused partial flocculation, resulting in increased FL of treated muds [21].

#### 2.3.3. Linear Expansion of Bentonite

**Figure 7:**
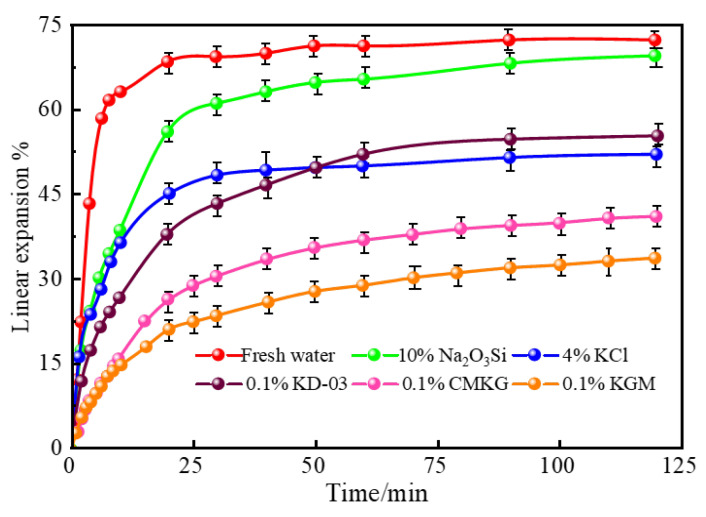
Curve of linear expansion rate versus time.

The swelling behavior of bentonite under different liquid media was investigated using a linear expansion meter. The test solutions included fresh water (control), 4.0% KCl, a 10% sodium silicate solution, and composite fluids containing 0.1% KD-03, KGM, and CMKG, respectively. The time-dependent linear expansion curves were obtained through 120 min of monitoring. Experimental results (Figure 7) demonstrated that the linear expansion rates of artificial core samples progressively increased with immersion time across all test fluids. After 120 min, the measured expansion rates were 72.1% (fresh water), 69.9% (10% sodium silicate), 52.1% (4% KCl), 55.4% (0.1% KD-03 gel), 34.0% (0.1% KGM gel), and 41.5% (0.1% CMKG gel). Compared with conventional oilfield inhibitors (KCl and sodium silicate), both KGM and CMKG exhibited superior inhibition performance against clay hydration swelling. At equivalent concentrations, KGM and CMKG gels demonstrated better anti-swelling efficiency than the heteropolysaccharide-based KD-03 inhibitor. This enhanced performance could be attributed to the abundant hydroxyl and sodium carboxylate groups on CMKG molecular chains, which effectively adsorb onto clay surfaces through hydrogen bonding interactions. This adsorption mechanism creates a protective barrier that impedes water-molecule penetration, thereby significantly suppressing clay hydration expansion [22].

#### 2.3.4. Mudball Experiments

**Figure 8:**
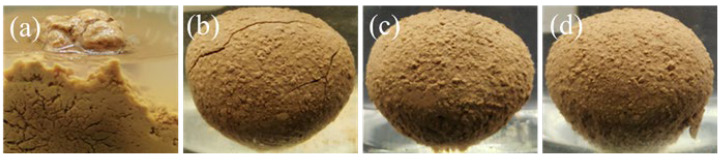
Appearance of mud balls after soaking in different solutions for 72 h. (**a**) water, (**b**) 4% KCl, (**c**) 0.3% KGM gel, (**d**) 0.3% CMKG gel.

Soak the mudballs (approximately 10 mg each) in tap water, 4% KCl, 0.3% KGM, and 0.3% CMKG colloidal solution, respectively. Record the macroscopic morphological evolution at intervals of 72 h. Figure 8 reveals that pellets in tap water underwent significant structural disintegration after 72 h immersion; the surface of the mud balls soaked in 4% KCl exhibited minor cracks, while those immersed in 0.3% KGM and CMKG showed no significant cracks. Both KGM and CMKG demonstrated prolonged inhibition effectiveness against clay hydration–expansion. The CMKG polymer chains establish a hydrogen-bond network between the hydroxyl groups (-OH) and the clay components, generating a coherent hydration adsorption film. This film sterically hinders the inflow of water, thereby inhibiting the hydration and dispersion of clay [23,24].

### 2.4. Application of KGM and CMKG in Fracturing Fluid

**Figure 9:**
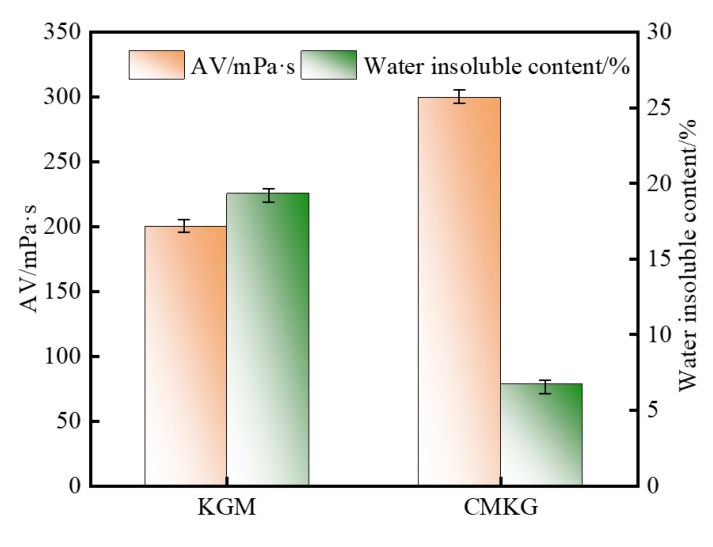
Basic parameters of KGM and CMKG fracturing fluid.

The CMKG was applied as a fracturing fluid thickener, and its performance was compared with KGM at 0.6% concentration in comparison. The measured parameters, including apparent viscosity and water-insoluble content, are presented in Figure 9. Both KGM and CMKG, as fracturing fluid thickeners, exhibit significant apparent viscosity. Compared to its pre-modified state, the apparent viscosity of the CMKG fracturing fluid increased by approximately 49.9%, while its water-insoluble content was reduced by 2.6%. These results demonstrate that CMKG effectively enhances the viscosity of KGM as a fracturing fluid thickener while reducing its water-insoluble components.

#### 2.4.1. Clay Compatibility

**Figure 10:**
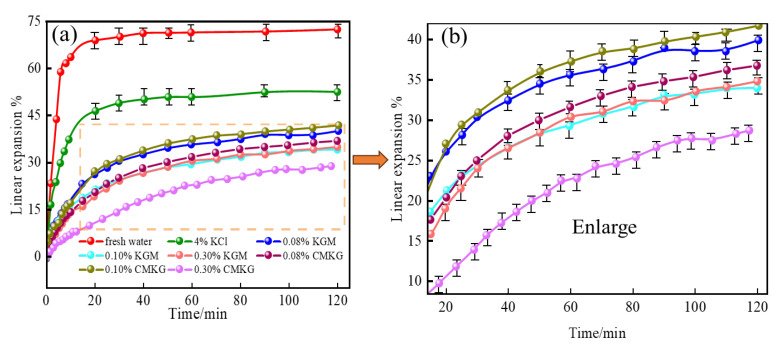
Effects of KGM and CMKG gels on the linear expansion rate of bentonite: (**a**) Global view from 0° to 120°. (**b**) Magnified view from 20° to 120°.

The compatibility between fracturing fluids formulated with 0.08%, 0.10%, and 0.30% KGM and CMKG gels and formation clay was evaluated through linear swelling rate measurements. As shown in Figure 10, compared with fresh water and 4% KCl solutions, both the pre- and post-carboxymethylation modified KGMs as fracturing fluid thickeners demonstrated good clay compatibility, indicating effective inhibition of formation clay hydration swelling and prevention of particle dispersion/migration. Furthermore, the clay linear swelling rate decreased progressively with increasing concentrations of KGM and CMKG. At a 0.3% dosage, the linear swelling rates for KGM and CMKG systems were 34.87% and 29.02%, respectively, representing a 16.8% reduction in swelling after modification. This enhanced clay swelling inhibition is attributed to the improved adsorption capacity of CMKG molecules onto clay surfaces through hydrogen bonding interactions between abundant hydroxyl and sodium carboxyl groups on the modified polymer chains and clay particles.

#### 2.4.2. Temperature Resistance Performance

**Figure 11:**
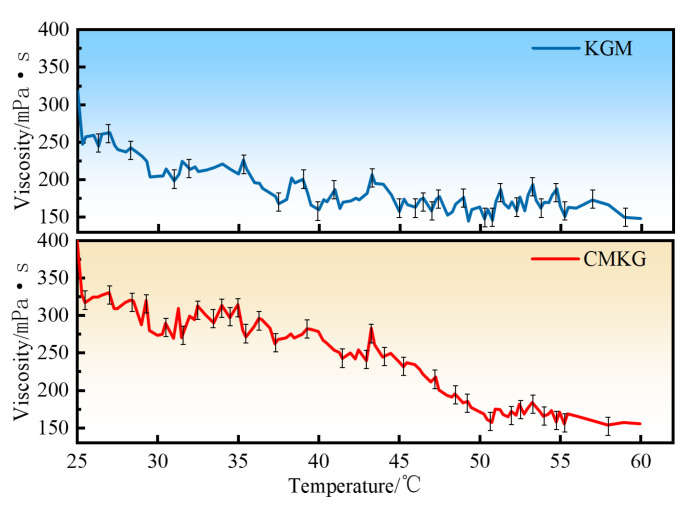
Viscosity–temperature curves of KGM and CMKG gels.

The thermal stability of fracturing fluids formulated with KGM and CMKG was evaluated, as shown in Figure 11. During hydraulic fracturing operations, maintaining adequate viscosity at formation temperatures is critical for operational efficiency [25,26]. Both KGM and CMKG-based fluids exhibited progressive decreases in apparent viscosity with increasing temperature, though with distinct degradation patterns. The CMKG fluid demonstrated notably higher initial viscosity followed by a sharp decline during early heating stages (20–50 °C), with viscosity reduction decelerating above 50 °C and subsequently maintaining a more uniform decline rate. In contrast, the KGM system showed moderate initial viscosity with relatively consistent viscosity–temperature behavior throughout the heating process. Notably, both systems maintained apparent viscosities above 150 mPa·s at 60 °C, indicating satisfactory thermal resistance for downhole applications. This sustained viscosity at elevated temperatures effectively mitigates the viscosity reduction rate of aqueous solutions under high-temperature conditions. Comparative analysis revealed the CMKG formulation demonstrated superior thermal stability, particularly in maintaining operational viscosity thresholds during the critical mid-temperature phase (40–60 °C). The enhanced performance is attributed to structural modifications improving macromolecular chain stability through reinforced intermolecular interactions [27,28] (Note: mean ± SD, *n* = 3).

#### 2.4.3. Shear Stability

**Figure 12:**
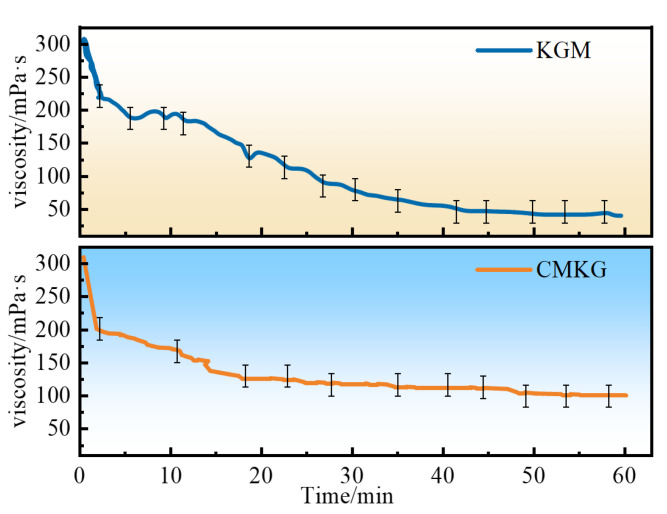
Viscosity variation curves of KGM and CMKG gels with temperature.

The shear rate was 170 s^−1^, and the initial temperature increased from 25 to 60 °C. The temperature was kept constant at 60 °C, and the shear continued for 3600 s. The viscosity curve is shown in Figure 12. With the progression of time, the viscosity of KGM shows a relatively significant downward trend, while the decrease in viscosity of CMKG is much smaller than that of KGM. CMKG can maintain a relatively high viscosity level in the later stage, which is more conducive to forming a stable gel system and maintaining the structural integrity of the gel. When the molecular structure of KGM is subjected to shearing or other actions, the molecular chains are prone to breakage, which disrupts the network structure of the gel it forms. This leads to a substantial decrease in its viscosity over time, making it unfavorable for the stable maintenance of the gel. In contrast, after modification, the molecular structure of CMKG changes. The introduced carboxymethyl groups and other modifications affect the intermolecular interactions, making the molecular chains less likely to break under similar conditions. This allows CMKG to better maintain the gel’s network architecture, thus preserving viscosity more effectively and reducing the degree of viscosity decline, which positively impacts the stability of gel properties. From the perspective of the overall change trend and the final stable viscosity, although KGM has a high initial viscosity, its viscosity decreases rapidly over time. Eventually, the viscosity of KGM drops to about 50 mPa·s, making it difficult to continuously support a good gel state. The viscosity of CMKG, however, drops to about 100 mPa·s. Obviously, the viscosity of CMKG is significantly higher than that of KGM (Note: mean ± SD, *n* = 3).

#### 2.4.4. Viscoelastic Evaluation

**Figure 13:**
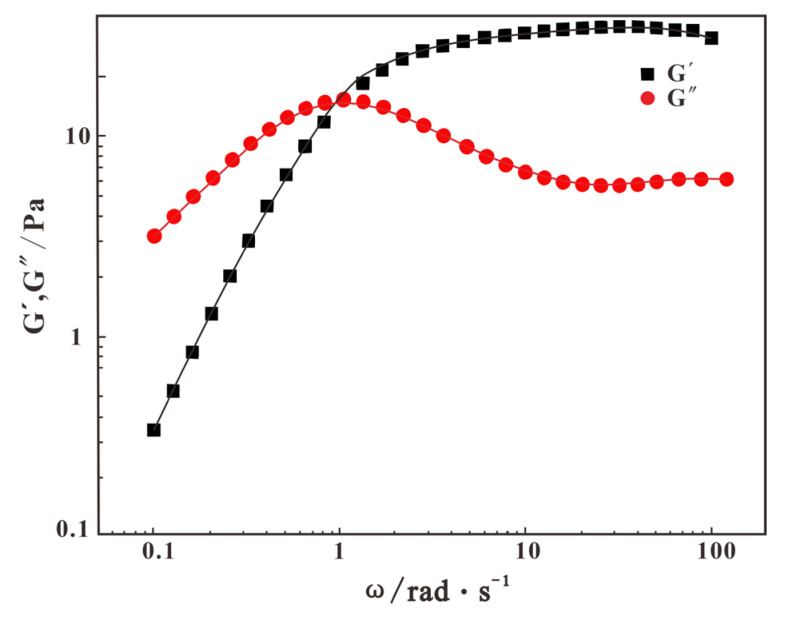
Variation curves of elastic modulus and viscous modulus of CMKG versus angular frequency.

The viscoelasticity of fracturing fluids is typically characterized by the elastic modulus (G′) and the viscous modulus (G″). The elastic modulus reflects the fluid’s elastic behavior, while the viscous modulus represents its viscous behavior. The variation curves of the elastic modulus G′ and viscous modulus G″ of CMKG at different stages with angular frequency ω are shown in Figure 13. As can be seen from the figure, in the low-frequency region, G″ > G′, indicating viscous dominance, and CMKG exhibits typical viscoelastic fluid behavior. In contrast, in the high-frequency region, G′ > G″, indicating elastic dominance, and CMKG demonstrates characteristics of a viscoelastic solid, which is a typical signature of the presence of wormlike micelles in the system. When the angular frequency ω = 1 rad·s^−1^, the elastic modulus and viscous modulus of the system are equal. Beyond this point, as the angular frequency continues to increase, G′ consistently exceeds G″. The elastic modulus gradually increases and shows reduced frequency dependence, while the system macroscopically exhibits a gel state. This phenomenon suggests the gradual formation of a spatial network structure within the system. As the angular frequency further increases, the elastic modulus stabilizes, indicating that this network structure becomes more compact.

#### 2.4.5. Gel-Breaking Performance

**Figure 14:**
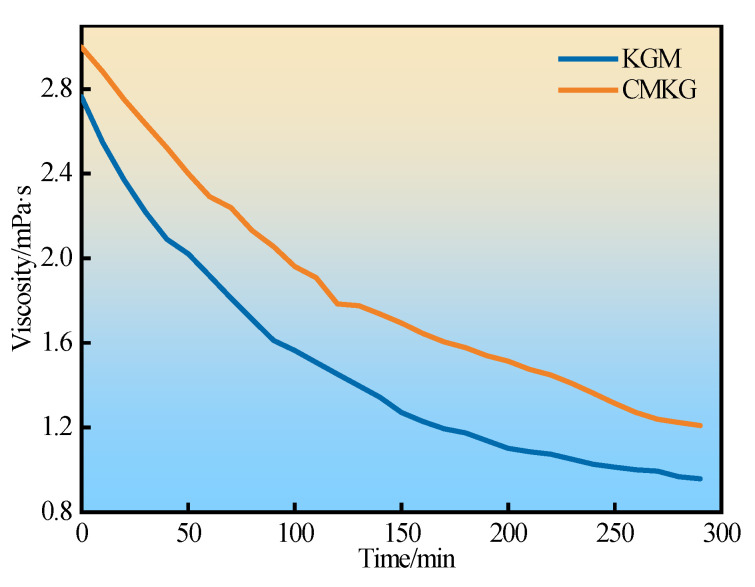
Curves of viscosity versus time for KGM and CMKG during gel breaking.

To meet the fundamental performance requirements of fracturing fluids, breakers must satisfy the following criteria: Firstly, the breaker should not prematurely reduce viscosity, ensuring the fracturing fluid maintains adequate performance for fracture propagation and proppant transport. Secondly, the gel-breaking time must be appropriately controlled. After entering the formation, the fracturing fluid must break down within a specific timeframe (viscosity < 5 mPa·s) to enable efficient flowback of the fluid system while retaining proppant in the fractures to maintain conductivity. Additionally, under the premise of achieving proper breakdown timing, the use of plant-gel fracturing fluids should minimize formation damage caused to the reservoir [29,30]. The gel-breaking performance of the KGM and CMKG fracturing fluid was tested at 95 °C using ammonium persulfate as the breaker. As the gel breaks, its three-dimensional network structure is compromised, which is directly manifested as a sharp decline in the system’s viscosity. This inverse correlation between the change and time is shown in Figure 14. The viscosity of the gel gradually decreased over time. The fracturing fluid achieved complete gel breakdown within 4 h, with no regelation phenomenon observed after cooling to room temperature. The viscosity of the broken fluid supernatant was close to that of water. Therefore, a dosage of 0.6% ammonium persulfate is sufficient to ensure rapid and effective gel breaking for the KGM and CMKG fracturing fluid system.

### 2.5. Environment-Friendly Evaluation

The environmental protection evaluation of CMKG was conducted using the microbial toxicity method, and the results are shown in the Table 7. As indicated in the table, CMKG exhibits a BOD_5_ value of 8345.5, a CODcr value of 13,562.7, and a BOD_5_/CODcr ratio of 0.61, all of which meet the environmental protection requirements. Additionally, the biological toxicity EC_50_ value is high, indicating low toxicity and compliance with environmental protection standards.

### 2.6. Mechanism Analysis

**Figure 15:**
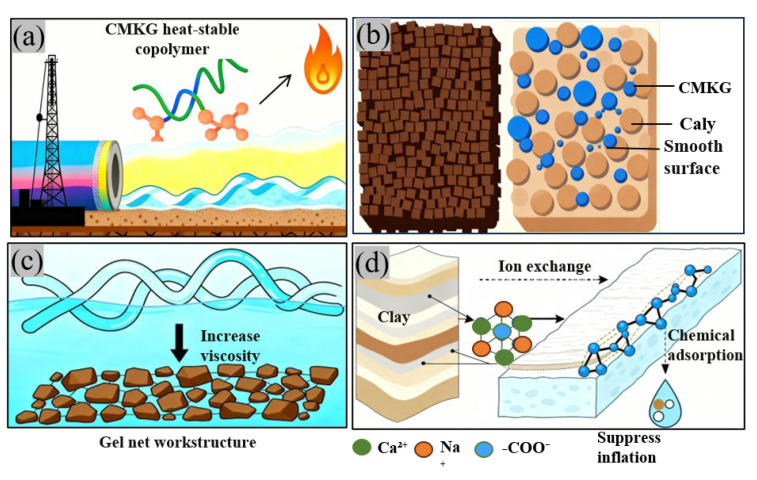
Schematic diagram of the action mechanism of CMKG as a drilling fluid additive: (**a**) High-temperature stability mechanism of CMKG; (**b**) formation of hydration film and lubrication mechanism; (**c**) CMKG entanglement and clay flocculation network mechanism; and (**d**) mechanism of inhibiting clay hydration.

As a drilling fluid additive, CMKG exerts key performance through its multi-dimensional action mechanisms in practical drilling operations. At the molecular level, under high-temperature conditions (Figure 15a), the polymer chains of CMKG are tightly coiled and crosslinked, forming a “reinforced network” that weaves the polymer chains into a stable three-dimensional network architecture. This effectively resists chain breakage and degradation caused by high temperatures, ensuring the drilling fluid maintains stable performance in high-temperature environments [31,32]. In terms of lubricity (Figure 15b), the abundant hydrophilic groups on its molecular chains undergo strong hydration in aqueous solutions, forming a hydrated film with a certain thickness and elasticity. The increase in drilling fluid viscosity stems from the dual network effect of polymer entanglement and clay flocculation (Figure 15c). On the one hand, CMKG molecular chains fully stretch and entangle in the aqueous solution to form a physical crosslinked network, increasing internal fluid resistance [33]. On the other hand, under alkaline conditions, the charge distribution characteristics of bentonite particles synergize with CMKG to construct a dense flocculation network structure, enabling precise regulation of the plastic viscosity and yield point of the drilling fluid. The core of inhibiting clay hydration expansion lies in double electric layer compression and interlayer confinement (Figure 15d). Functional groups such as carboxyl and hydroxyl groups on CMKG tightly bind to active sites on the surface of clay particles through hydrogen bonding, electrostatic adsorption, etc., forming a dense, inhibitory protective film. This effectively blocks water molecules from entering the clay interlayers, suppresses clay hydration expansion and dispersion.

## 3. Conclusions

In conclusion, we have used dichloroacetic acid as the modifying agent and proposed a carboxymethylated modification method for KGM. The CMKG can be easily prepared within an alkaline environment utilizing an ethanol/water (3:1) dispersion medium. The performance of CMKG as an additive in drilling fluids and fracturing fluids was systematically evaluated. When employed as an additive in drilling fluids, the incorporation of 0.3% (by weight) CMKG resulted in a substantial 418.2% increase in the system’s viscosity. Concurrently, it enhanced the lubrication performance of the filter cake and improved the thermal stability of the drilling fluid system, enabling it to withstand temperatures up to 120 °C. In terms of inhibiting clay hydration, CMKG exhibits excellent performance compared with 4% potassium chloride and 10% sodium silicate solutions, which are derived from the wrapping and plugging effects of carboxyl and hydroxyl groups on clay particles through hydrogen bonding and chemical adsorption [34]. After a 120 min exposure period, the linear expansion rate of bentonite in the presence of CMKG was measured at a mere 34.07%, indicating its effective inhibition capability. In the context of fracturing fluid applications, a 0.6% CMKG solution exhibited an apparent viscosity of 299.42 mPa·s and significantly reduced the content of water-insoluble substances to 16.7%. Following continuous shearing at 60 °C for 60 min, the solution maintained a viscosity in excess of 60 mPa·s. Li Qiang et al. [35] demonstrated that the high viscosity and excellent shear resistance of fracturing fluids contribute to enhancing the fracturing effect, enabling fractures to extend effectively and remain open. The performance indicators of CMKG fully meet the requirements of modern fracturing processes. These results collectively suggest that CMKG effectively enhances the rheological properties of mud, exhibiting notable viscosity-increasing and filtration-reducing effects. This research expands the application scope of KGM, reduces reliance on traditional additives, and promotes the greening of additives in petroleum engineering. Furthermore, we provide a novel and environmentally friendly treatment agent, which is conducive to enhancing the efficiency of oil exploitation and reducing costs.

## 4. Materials and Methods

### 4.1. Materials

Analytically pure NaOH was purchased from Zhengzhou Panni Chemical Reagent Factory; glacial acetic acid (≥99.5%) was from Tianjin Tianli Chemical Reagent Co., Ltd. (Tianjin, China); absolute ethanol (99%) was from Tianjin Hongyan Reagent Factory (Tianjin, China); analytical grade dichloroacetic acid (DCA) was acquired from Tianjin Tianli Chemical Reagent Co., Ltd. (Tianjin, China); analytically pure Konjac gum (KGM) and bentonite were obtained from Xi’an Yongjiu Chemical Co., Ltd. (Xi’an, China); and KD-03 inhibitor was sourced from Tangshan Jidong Ruifeng Chemical Co., Ltd. (Tangshan, China).

SD6 medium-pressure filtration loss analyzer and an NP-1 ambient-pressure swelling tester with industrial were supplied by Qingdao Haitongda Specialized Instrument Factory (Qingdao, China); the NZ-3A viscometer and WSN-1 Ubbelohde viscometer were acquired from Ningbo Tianheng Instrument Factory (Ningbo, China); the Nicolet 5700 FTIR spectrometer was purchased from Thermo Fisher Scientific, Waltham, MA, USA; a TGA/DSC1 thermogravimetric analyzer was acquired from Mettler Toledo, Columbus, OH, USA; the GGS71-A DSC was obtained from Mettler Toledo, Greifensee, Switzerland; and the VarioMICRO elemental analyzer was purchased from Elementar, Langenselbold, Germany. The Haake RS6000 rheometer was purchased from Anton Paar China (Shanghai, China).

### 4.2. Modification of KGM

**Figure 16:**
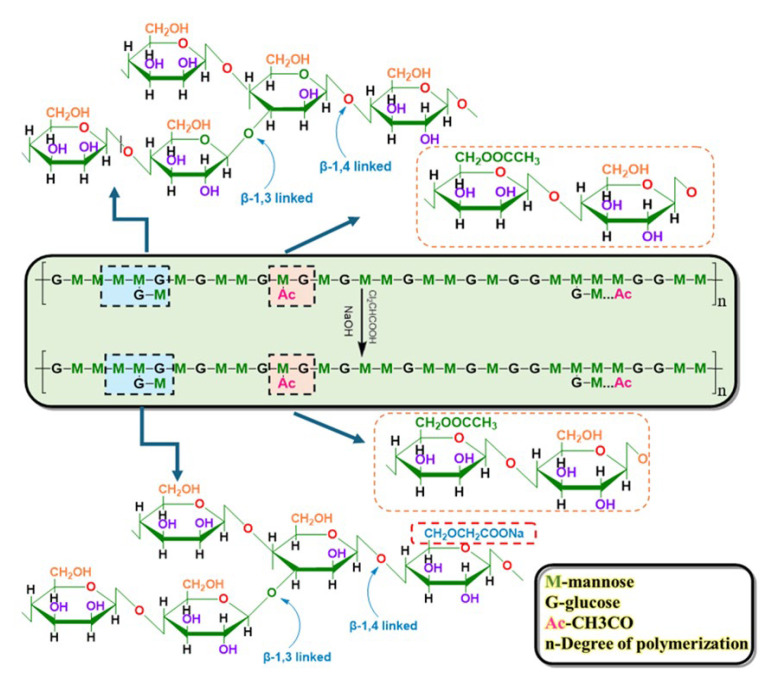
Modification of KGM.

Weigh KGM powder and transfer it into a round-bottom flask equipped with a reflux condenser, adding water and absolute ethanol simultaneously. Initiate magnetic stirring and reflux. While stirring, slowly add NaOH and DCA to the system. Subsequently, conduct the reaction under alkaline conditions in a constant-temperature water bath with mechanical stirring for a specified duration. Chloroacetic acid molecules undergo ionization to exist in the form of carboxymethyl anions (-CH_2_COO^−^). The alkoxy anion in the KGM molecule, due to its strong nucleophilicity, attacks the carbon atom connected to the chlorine atom in the chloroacetic acid molecule, initiating a nucleophilic substitution reaction (Figure 16). As the reaction proceeds, the chlorine atom departs as a leaving group, and the carboxymethyl group is securely attached to the hydroxyl positions of the glucose and mannose residues in the KGM molecule through a covalent bond. Upon completion, glacial acetic acid was used to adjust the system pH to neutral. The product was then washed with anhydrous ethanol and isolated via suction filtration. Finally, the modified product was air-dried at ambient temperature to yield CMKG, which was stored in a sealed bag for future use.

### 4.3. Orthogonal Experiment

To systematically investigate the influence of reaction parameters on CMKG, an L9 (3^4^) orthogonal array was designed, with the viscosity and water-insoluble content of the modified product as key evaluation metrics. The orthogonal experiments examined four critical factors: reaction temperature, duration, NaOH dosage, and DCA dosage. The experimental factors and their corresponding levels are summarized in Table 8, while the L9 (3^4^) orthogonal test matrix is detailed in Table 9.

### 4.4. Characterization Methods

Thermal behavior analysis was performed using differential scanning calorimetry (DSC, Model GGS71-A) under a nitrogen atmosphere (50 mL/min flow rate) at a heating rate of 10 °C/min from 25 to 500 °C. indium calibration and an empty aluminum crucible are applied as the reference. Fourier transform infrared (FTIR) spectroscopy was conducted on a Nicolet 5700 spectrometer. Dried samples were mixed with KBr at a 1:100 ratio and pelletized for analysis in transmission mode at a resolution of 2 cm^−1^ with 64 scans. TGA analysis was carried out on a TGA/DSC1 instrument under nitrogen protection (20 mL/min flow rate), where 10 mg samples in alumina crucibles were heated synchronously to 500 °C. Elemental composition (C/H/N/S/O) was determined using a VarioMICRO analyzer via high-temperature combustion (950 °C under oxygen flow), with quantification based on acetanilide standard curves.

### 4.5. Drilling Fluid Performance Evaluation

#### 4.5.1. Temperature Resistance Test

The drilling fluid was prepared by adding 0.2% sodium carbonate (pre-hydrated for 5 min) and 4% calcium-based bentonite in water, while stirring at high speed for 2 h. Base slurry was prepared with clear water as the medium. Then, the solution was aged at room temperature for 24 h. Subsequently, the base slurry was re-stirred for 5 min, drilling fluid additives were gradually added, continuous high-speed shearing was carried out for 20 min, and it was finally sealed and aged for 16 h to optimize performance stability. To determine the temperature resistance performance of KGM/CMKG, water-based drilling fluids with drilling fluid additives were placed at different aging temperatures (30 °C, 60 °C, 90 °C, 120 °C, and 150 °C) for 16 h, respectively. The main properties of the aged drilling fluid—including apparent viscosity (AV), plastic viscosity (PV), dynamic shear force (YP), static fluid loss at 7.5 min (FL), and filter cake friction resistance (tg)—were determined.

#### 4.5.2. Linear Swelling

Load oven-dried bentonite into the core mold assembly. Compress the assembly at 10 MPa for 5 min using a hydraulic press to form core discs. Prepare KGM and CMKG solutions at predetermined concentrations. Measure disc swelling in these solutions with a linear swell meter, recording expansion values at timed intervals. Calculate the linear expansion rate of the clay.

Sr = Ro/∆L × 100%(1)

where Sr—linear swelling rate of bentonite; Ro—swelling of bentonite, mm; and ∆L—core thickness, mm.

#### 4.5.3. Mud Ball Test

Aqueous solutions of KGM and CMKG were prepared at specified concentrations and transferred into beakers of identical volume. Sodium bentonite was homogeneously mixed with distilled water at a mass ratio of 2:1, followed by manual kneading to form multiple mud balls (approximately 10 g each). These mud balls were gently immersed in separate beakers containing equivalent volumes of test solutions or distilled water (control group). Surface morphological alterations of the mud balls were systematically observed and photo documented at predetermined time intervals to evaluate their structural stability and hydration inhibition characteristics.

### 4.6. Fracturing Fluid Performance

#### 4.6.1. Rheological Property Test

Weigh 3.0 g of KGM and CMKG and add them to 500 mL of distilled water. Stir at high speed for 20 min to dissolve. Then place the solution in a water bath at 30 °C for 4 h. After the viscosity stabilizes, measure the apparent viscosity, water-insoluble matter, and crosslinking property. The gel solutions of KGM and its derivatives were then loaded into the rheometer test cup. First, a temperature scanning test was conducted (at a shear rate of 10 s^−1^, heated from 1 °C/min to 60 °C, and the change in apparent viscosity with temperature was recorded). Subsequently, the gel was subjected to continuous shearing at a shear rate of 170 s^−1^ for 1 h, maintaining a temperature of 30 °C. The curve of viscosity change over time was monitored to evaluate the thermal stability and shear tolerance of the gel.

#### 4.6.2. Viscoelasticity Assessment

The tests were conducted using a HAAKE rheometer equipped with a CP50-1 rotor in oscillation mode. The specific procedure was as follows: first, the temperature was set to 25 °C, followed by a strain sweep to determine the linear viscoelastic region (LVR) of the system. Within the linear viscoelastic region, the strain parameter was fixed, and a frequency sweep was performed over a range of 0.01 rad/s to 100 rad/s. This test allowed for the investigation of the variation of the elastic modulus (G′) and viscous modulus (G″) with angular frequency.

#### 4.6.3. Determination of Gel-Breaking Performance

Take 50 mL of KGM and CMKG containers and conduct the gel-breaking reaction at a constant temperature (oil layer temperature). Collect the supernatant at regular intervals to measure the apparent viscosity to evaluate the gel-breaking rate and the stability of the gel solution.

## Figures and Tables

**Table 1:** Analysis of orthogonal experimental range (insoluble matter content).

Subjects	a	b	c	d
K1	21.27	27.73	18.57	16.67
K2	28.00	18.57	25.97	18.33
K3	18.27	21.23	23.00	32.53
Range	9.73	9.17	7.40	15.87
Arrange in order	2	3	4	1
Optimization condition	a2	b1	c2	d3

**Table 2:** Orthogonal test results.

Experiment No.	0.6% Apparent Viscosity of Solution mPa·s	Water Insoluble Matter Content %	Crosslinkability
Bare	199.62	19.31	pickable
1	299.42	16.70	pickable
2	233.88	16.60	pickable
3	98.32	30.50	pickable
4	226.44	46.70	pickable
5	65.73	18.70	pickable
6	274.11	18.60	pickable
7	32.17	19.80	pickable
8	294.97	20.40	pickable
9	205.58	14.60	pickable

**Table 3:** Analysis of orthogonal experimental range (apparent viscosity).

Subjects	a	b	c	d
K1	210.54	186.01	289.50	190.24
K2	188.76	198.19	221.97	180.05
K3	177.57	192.67	65.41	206.58
Range	32.97	12.18	224.09	26.52
Arrange in order	2	4	1	3
Optimization condition	a1	b2	c1	d3

**Table 4:** Elemental Analysis of KGM and CMKG.

Sample	Element % (Mass)				
	C	H	N	S	O
KGM	37.80	6.16	0.62	0.33	55.09
CMKG	39.37	6.41	0.18	0.31	53.72

**Table 5:** Influence of KGM and CMKG addition amount on drilling fluid performance.

Additive	AV/ mPa·s	PV/mPa·s	YP/ Pa	YP/PV/ Pa/mPa·s	FL/ mL	tg
Mud	5.50	4.00	1.40	0.36	13.30	0.0963
Mud + 0.05%KGM	12.50	7.50	4.80	0.64	12.10	0.0437
Mud + 0.10%KGM	24.00	15.00	8.60	0.58	18.50	0.0699
Mud + 0.30%KGM	27.20	10.00	16.30	1.63	52.00	-
Mud + 0.05%CMKG	16.10	9.50	6.20	0.65	12.70	0.0875
Mud + 0.10%CMKG	25.70	9.50	10.80	1.14	18.80	0.0875
Mud + 0.30%CMKG	28.50	11.00	11.00	1.01	21.30	0.0963

Note: “-” indicates that the mud cake cannot be removed.

**Table 6:** Influence of temperature on drilling fluid performance.

T/°C	Additive	AV /mPa·s	PV /mPa·s	YP /Pa	YP/PV /Pa/mPa·s	tg	FL /mL
30	Mud	5.50	4.00	1.40	0.36	0.0963	13.30
	KGM	24.00	15.20	8.64	0.58	0.0699	18.50
	CMKG	22.50	11.00	11.04	1.01	0.0963	21.30
60	Mud	3.40	2.50	0.34	0.14	0.1152	13.50
	KGM	13.50	7.00	6.24	0.89	0.0787	12.00
	CMKG	17.50	8.00	9.12	1.14	0.0699	17.00
90	Mud	2.50	2.10	0.05	0.02	0.1584	14.80
	KGM	8.20	5.00	2.88	0.58	0.0524	11.50
	CMKG	10.50	6.20	4.13	0.66	0.0612	14.20
120	Mud	2.70	2.80	0.10	0.04	0.2315	16.40
	KGM	4.00	3.50	0.48	0.14	0.0699	12.40
	CMKG	4.90	3.80	1.06	0.13	0.0524	13.00
150	Mud	3.50	3.20	0.20	0.06	0.2168	17.00
	KGM	3.80	2.80	0.35	0.12	0.0699	14.20
	CMKG	4.50	3.60	0.41	0.11	0.0524	15.20

**Table 7:** Detection data of CMKG environmental performance.

Environmental Performance Indicators	EC_50_	BOD_5_	COD_Cr_	BOD_5_/COD_Cr_
Index Indicator	21,400	8345.5	13,562.7	0.61

**Table 8:** Level factors of orthogonal test.

Level	A: Temperature/°C	B: Time/min	C: Additions (ω/%)	**D:** **Additions** **(ω/%** **)** **(DCA/KGM** **)**
			(Sodium Hydroxide NaOH/KGM)	
1	30	2	0.1%	0.1%
2	50	4	1.0%	0.5%
3	70	6	2.0%	1.0%

**Table 9:** L9 (3^4^) orthogonal experimental design.

Experiment No.	A	B	C	D
1	1	1	1	1
2	1	2	2	2
3	1	3	3	3
4	2	1	2	3
5	2	2	3	1
6	2	3	1	2
7	3	1	3	2
8	3	2	1	3
9	3	3	2	1

## Data Availability

Data will be made available on request.

## Abbreviations

The following abbreviations are used in this manuscript:

KGM	Konjac gum
CMKG	Carboxymethylated Konjac gum
DCA	Dichloroacetic acid
